# Teacher Well-Being in EFL/ESL Classrooms

**DOI:** 10.3389/fpsyg.2021.732412

**Published:** 2021-08-04

**Authors:** Zhong Li

**Affiliations:** School of Foreign Languages, Xinyang College, Xinyang, China

**Keywords:** EFL/ESL classrooms, teacher well-being, positive psychology, working conditions, second language

## Abstract

A popular point in the last 20 years in academic and business settings is well-being at work that is in line with positive psychology, through which one can understand how to make working conditions enjoyable. Alternatively, teaching has been recognized as the most stressful career. Although, not many studies in the form of review have been carried out to focus on the notion of the well-being of teachers in English as a second language (ESL) or English as a foreign language (EFL). According to the literature review, the definition of this construct, the factors related to it, and the empirical studies in this domain are presented. In conclusion, the implications of well-being for teachers, school principals, teacher-trainers, and future researchers are provided, and new directions for future research are delineated.

## Introduction

The primary objectives of the educational framework are, undoubtedly, successful education, which covers different intricacies and is influenced by various variables. A bulk of research substantiates the possibility that teacher psychology is one of these variables (e.g., Van Horn et al., 2004; Acheson et al., 2016). When it comes to the language-learning psychology field, despite the great consideration of the psychology of the learners, moderately, the psychology of the teachers was not taken into consideration, which is as indispensable as the former in the language class (Mercer et al., 2016). In the last two decades, the developing popularity of positive psychology (PP) has caused an incredible shift away from the restrictive emphasis on issues in general psychology (Dewaele et al., 2019). Due to a call for a greater prominence on the positive aspect of life, PP has gained great momentum that considers human well-being and has investigated how individuals can work hard and how they can “thrive and flourish” (MacIntyre and Mercer, 2014, p. 154).

In addition, theories of PP in language acquisition study were adopted recently, and as it was stated by Mercer and MacIntyre (2014) that Lake (2013) was the first who presented PP perception in language acquisition, and with the advent of PP in this field, there has been a change from negative (paucities) to strong points (positive) (MacIntyre, 2016). It is proved that positive emotions are associated with attitudes toward the learning circumstances, the teacher and the course, and motivation to acquire English (Dewaele and Li, 2020). Well-being is an active construct of PP which refers to the way a person thinks, perceives, and circumstances at a prearranged period (Greenfield, 2015). When talking about well-being in PP, the construct of flourishing (having an enthusiasm for life) is often taken into account (Kern et al., 2016). In the English as a foreign language (EFL) setting, teacher well-being may also be endangered by numerous issues, such as deficient language skills, lack of linguistic knowledge, the absence of confidence (Mousavi, 2007; Talbot and Mercer, 2018), and the stress imposed on them due to a heavy workload (Clipa and Boghean, 2015). Other constructs that support teacher well-being are resilience and self-efficacy. So, it is proposed that flourishing is not contradicted to the manifestation of stress in the lives of people but instead changes the viewpoints toward the positive qualities that assist a person in managing difficulties (Greenfield, 2015).

So far, most researchers have broadly focused on negative emotions to examine their adverse impacts on the language learning process (e.g., Saboori and Pishghadam, 2016; Özdemir and Demir, 2019); however, positive emotions have been ignored in most academic research and little attention has been given to the changes that they can make to English as a second language (ESL)/EFL classes as Lantolf and Swain (2019) stated that the role of positive emotions is increasing across existing limitations. Furthermore, several studies have been conducted to focus on teacher well-being; however, still limited evidence is presented on the history, definitions, and determinants of well-being relevant for ESL/EFL teachers, which seems to be necessary for language-learning quality (Weiland, 2021). As a result, to reinforce the emergent attention to PP in ESL/EFL classes and teaching inquiries, in this review article, the researcher tried to clarify the well-being of teachers, factors affecting it, and the previous studies which have been carried out in this domain.

## The Concept of the Well-Being of the Teacher

Butler and Kern (2016) referred to several theories developed by PP scholars to define the well-being construct. The study of well-being was in two systems, subjective well-being and psychological well-being (PWB), measured by hedonic and eudemonic methods, respectively (Keyes, 2002). Even though both have values in research on the well-being of teachers, the hedonic perspective, especially apparent in research on “subjective well-being,” (Diener and Lucas, 2000) contends that well-being fixates principally on expanding delight and diminishing agony, and will, underscore outer impacts on sensations of well-being instead of inner sources, including inspirations, yearnings, and wants of the teachers. As opposed to the hedonic perspective, the eudaimonic one deals with human thriving, which is characterized as a psychosocial development that incorporates fulfillment and good connections, feeling skillful and certain, and accepting that life is significant and intentional and underscores both satisfaction and seriousness (Diener et al., 2010). Flourishing is the objective of PP that refers to well-being using great levels of PERMA in life, which as stated by Seligman (2018), signifies positive emotion (P), engagement with actions that consider powers of the individual (E), increasing constructive personal relationships (R), finding meaning by helping a reason further than oneself (M), and identifying parts of achievement and success (A). Through this model, well-being has arisen from the relations of positivity in all these elements, which refers to the eudemonic view of wellbeing (Mercer and Gregersen, 2020). When it comes to teaching, well-being has been investigated concerning satisfaction of the teachers with their positions and the feelings that are brought about by their professional experiences (Margolis et al., 2014; Collie et al., 2016).

The expansion of studies on well-being was proposed by Diener (2009), containing the area of work that refers to the healthy functioning of an individual in their work setting. Coleman (2009) makes the significance of well-being for the teaching career clear when contending that barely does it make sense to handle the emotional health of students in a school without taking care of the emotional health of the staff. The well-being of teachers is noteworthy because of its results for learners and schools, for example, teachers encountering low well-being in the working environment might be less useful and be more inclined to leave work (Boyd et al., 2006).

### Factors Affecting the Well-Being

Well-being has several dimensions that are dependent upon a collection of internal and external aspects (Benevene et al., 2018). The aspects which affect teacher well-being are either positive or negative (Zadworna-Cieślak and Karolina, 2018). Among negative aspects, stress and burnout can play key roles, which were studied earlier regarding negative emotions of teachers (Capone et al., 2019; Derakhshan et al., 2021).

As Ryan and Deci (2001) postulated, occupational well-being among teachers, which is related to ideal mental capacity and their positive work experience, has been characterized by the existence of positive dimensions, such as work fulfillment and interests in work (Benevene et al., 2018). In addition, self-efficacy is the first element which is a fundamental piece of well-being that may influence self-inspiration as well as both personal and professional life directions (Bandura, 2000). Furthermore, the self-efficacy of the teacher is characterized by the self-decisions of teachers concerning their capability of influencing learner results, particularly of learners who seem unmotivated or who are hard to instruct (Ross et al., 2012).

Besides, the other positive factors or strategies to deal with stress, which subsequently affect well-being, are resilience, mindfulness, and emotional regulation (Lovewell, 2012; Zadworna-Cieślak and Karolina, 2018). Resilience is a fundamental piece of human life (Bullough, 2011), whereas mindfulness refers to an ability to attend freely to what is occurring in the ongoing moment experience of an individual (Creswell and Lindsay, 2014), and emotional regulation is the capability a person to control when and how to encounter emotions in the activity they are interested in Yin et al. (2016). They are both regarded as the conditions of supporting good teaching in challenging conditions to manage stress and promote wellbeing (Bullough, 2011; Garland et al., 2011). As a result, teachers with high degrees of stress and burnout, and without satisfactory managing abilities and strategies, such as resilience, mindfulness practices, and emotional regulation, were not successful in their job and consequently cannot fulfill the accomplishment of their students (Herman et al., 2017).

## Empirical Studies

Greenier et al. (2021) proved the effect of emotional regulation and PWB as predictors of work engagement in both British and Iranian teachers, and the results indicated that both variables predicted work engagement for the participants, even though the association of the PWB and work engagement was more resilient for British teachers. Numerous studies (e.g., Svence and Majors, 2015; Brouskeli et al., 2018) have revealed a relationship between resilience and the well-being of teachers. In addition, a study by Barbieri et al. (2019) evinced how positive insight of teachers on the working conditions may improve a significant level of well-being at work, and how these aspects are in line with the job satisfaction of the teachers. Besides, a study by Han et al. (2020) examined the relationships among challenging job demands and resources (JD-R) and the effect of the efficacy of teachers among university professors. These findings proved the effective use of the JD-R model and the impact of teacher efficacy on the correlation between the professional features and the well-being of the teachers.

## Implications and Future Directions

Based on this mini-review that focuses on the well-being of the teacher as positive emotions of the teacher, many pedagogical implications were provided for different groups in EFL/ESL settings, such as teachers, school principals, teacher-trainers, and researchers.

The essence of any teaching organization is its teachers, and their well-being should be dominant in the educational context not only to improve the ability of the learners but also to motivate and encourage them to participate in classroom activities. Teachers seek an approach that does not stress them and would give them more scope to apply creative teaching methods and give interesting exercises. Well-being and the remarkable difficulties that teachers face in guaranteeing health in an EFL/ESL setting is still a significant thought to better maintain and support teachers in their expert work. The trainers of the teachers should be educated regarding PP, and schools should teach well-being strategies, such as resilience, mindfulness, and emotional regulation alongside scholarly ones through proficient improvement to their teachers to help lighten the inescapable pressure and stress that are met in their profession. Besides, school principals need to provide a healthy setting for their teachers to support their well-being that can foster a successful classroom. The present review is set to raise the prominent role of PP, in general, and the well-being of teachers, in particular, to encourage teachers and researchers to bear these issues in mind and give better ideas for the progress in this field, and, accordingly, contribute to their awareness of the mechanism for establishing well-being among EFL/ESL teachers.

## Author Contributions

ZL read the relevant literature and presented the implications of well-being for the research in SLA.

## Conflict of Interest

The author declares that the research was conducted in the absence of any commercial or financial relationships that could be construed as a potential conflict of interest.

## Publisher’s Note

All claims expressed in this article are solely those of the authors and do not necessarily represent those of their affiliated organizations, or those of the publisher, the editors and the reviewers. Any product that may be evaluated in this article, or claim that may be made by its manufacturer, is not guaranteed or endorsed by the publisher.
